# The Degradative Capabilities of New *Amycolatopsis* Isolates on Polylactic Acid

**DOI:** 10.3390/microorganisms7120590

**Published:** 2019-11-20

**Authors:** Francesca Decorosi, Maria Luna Exana, Francesco Pini, Alessandra Adessi, Anna Messini, Luciana Giovannetti, Carlo Viti

**Affiliations:** 1 Department of Agriculture, Food, Environment and Forestry (DAGRI)—University of Florence, Piazzale delle Cascine 18, I50144 Florence, Italy; francesca.decorosi@unifi.it (F.D.); lunaexana@gmail.com (M.L.E.); francesco.pini@unifi.it (F.P.); alessandra.adessi@unifi.it (A.A.); anna.messini@unifi.it (A.M.); luciana.giovannetti@unifi.it (L.G.); 2 Genexpress Laboratory, Department of Agriculture, Food, Environment and Forestry (DAGRI)—University of Florence, Via della Lastruccia 14, I50019 Sesto Fiorentino, Italy

**Keywords:** polylactic acid, polylactide, *Amycolatopsis*, biodegradation

## Abstract

Polylactic acid (PLA), a bioplastic synthesized from lactic acid, has a broad range of applications owing to its excellent proprieties such as a high melting point, good mechanical strength, transparency, and ease of fabrication. However, the safe disposal of PLA is an emerging environmental problem: it resists microbial attack in environmental conditions, and the frequency of PLA-degrading microorganisms in soil is very low. To date, a limited number of PLA-degrading bacteria have been isolated, and most are actinomycetes. In this work, a method for the selection of rare actinomycetes with extracellular proteolytic activity was established, and the technique was used to isolate four mesophilic actinomycetes with the ability to degrade emulsified PLA in agar plates. All four strains—designated SO1.1, SO1.2, SNC, and SST—belong to the genus *Amycolatopsis*. The PLA-degrading capability of the four strains was investigated by testing their ability to assimilate lactic acid, fragment PLA polymers, and deteriorate PLA films. The strain SNC was the best PLA degrader—it was able to assimilate lactic acid, constitutively cleave PLA, and form a thick and widespread biofilm on PLA film. The activity of this strain extensively eroded the polymer, leading to a weight loss of 36% in one month in mesophilic conditions.

## 1. Introduction

Bioplastics—biodegradable plastic materials synthesized from biomass sources—are considered an attractive alternative to conventional petroleum-based polymers, which have become one of the most serious environmental issues because of their extensive use, incorrect disposal, and non-degradability. Among bioplastics, polylactic acid (PLA), an aliphatic polyester synthesized from lactic acid, can be efficiently produced by the fermentation of starch feedstock [1]. PLA is a bioplastic with a broad range of applications because of its excellent proprieties, such as a high melting point, a high mechanical strength, a high degree of transparency, and ease of fabrication. It has been extensively used in the biomedical field as a material for sutures and bone fracture fixation, as well as for packaging materials and mulching films. PLA production is predicted to reach at least 800,000 tons worldwide by 2020 [2]. Consequently, the safe disposal of PLA waste is an emerging environmental problem since its biodegradation generally requires a long time, ranging from several months to years [3]. PLA biodegradation is a complex process that occurs in three phases [4]: biodeterioration, biofragmentation, and assimilation. Biodeterioration encompasses the physical and chemical modifications of PLA properties after a microbial community adheres to the material surface as a biofilm; biofragmentation is the cleavage of PLA into oligomers, dimers, or monomers by extracellular hydrolytic enzymes; assimilation involves the simple molecules resulting from biofragmentation being transported to the cytoplasm of microbial cells and catabolized.

In natural soil environments, PLA is less susceptible to biodegradation than other aliphatic polyesters because PLA-degrading microorganisms are not widely distributed in soils and present at very low percentages compared with microorganisms able to attack other biodegradable plastics such as poly-ε-caprolactone (PCL) or polyhydroxybutyrate (PHB) [5]. To date, a limited number of PLA-degrading bacteria have been isolated. Most of them are “rare actinomycetes” belonging to the genera *Amycolatopsis*, *Lentzea*, *Kibdelosporangium*, and *Streptoalloteichus* (Pseudonocardiaceae); *Thermomonospora* and *Actinomadura* (Thermomonosporaceae); *Laceyella* and *Thermoactinomyces* (Thermoactinomycetaceae); *Micromonospora* (Micromonosporaceae); and *Thermopolyspora* (Streptosporangiaceae) [2,5,6]. Rare actinomycetes are usually regarded as actinomycetes whose isolation frequency is much lower than that of streptomycetes [7]. Interest in rare actinomycetes has increased because they are believed to be an essential source of bioactive molecules [8]. Furthermore, this group of microbes can produce extracellular enzymes such as amylase, chitinase, cellulase, glucanase, and protease that hydrolyze molecules characterized by complex structures [9]. Less frequently, strains outside the actinomycete group have been identified as PLA degraders, such as strains belonging to the genera *Pseudomonas* and *Stenotrophomonas* [10,11]. Moreover, PLA-degrading activity has been reported for several members of the Bacillaceae family, including *Brevibacillus* sp. [12], *Bacillus smithii* [13], *Geobacillus* sp. [14], and *Bacillus licheniformis* [15].

Researchers have primarily focused their attention on the isolation of thermophilic microorganisms because PLA undergoes rapid chemical hydrolysis at elevated temperatures, which facilitates the microbial mineralization of PLA [10]. However, the role of microorganisms in the PLA degradation process is still poorly understood since, in the environment, a combination of abiotic and biotic factors contributes to biodegradation. Given the conditions in most environments, the degradation of PLA waste in nature is largely due to the activity of mesophilic bacteria [10]. Thus, the isolation and selection of mesophilic strains with the ability to efficiently degrade PLA are considered a promising strategy for the development of a biotechnological process for PLA biodegradation in environmental conditions.

Different approaches have been used to isolate potential PLA-degrading microorganisms, but all have resulted in a very low frequency of isolation. Therefore, several environmental samples need to be tested to obtain a few potential PLA degraders [14,16,17,18]. The PLA-degrading ability of bacteria is generally evaluated by using a clear zone assay [19] that is typically applied to (i) bacteria directly extracted from natural environmental samples (e.g., soils, compost, active sludge) [16,20] and (ii) bacteria obtained from enrichment cultures produced by inoculating a PLA-containing mineral medium with microorganisms from environmental samples [14,17,21]. The test entails the cultivation of bacteria on emulsified PLA agar plates that are opaque. After incubation, colonies surrounded by a clarification halo (due to PLA hydrolysis by extracellular depolymerases) are considered potential PLA degraders. The actual ability of these strains to degrade PLA must be verified by further experiments that evaluate their ability to deteriorate the solid polymer.

In this work, an improved isolation procedure was developed with the aim of isolating and characterizing new rare mesophilic PLA-degrading actinomycete strains for use in bioaugmentation biotechnological projects.

## 2. Materials and Methods

### 2.1. Materials

PLA 4032D with about 2% d-lactide (NatureWorks, Minnetonka, MN, USA) was used in the experiments. The average molecular weight (Mw) ranged from 190 to 230 KDa [22].

### 2.2. Bacterial Strains and Media

#### 2.2.1. Strains

*Lentzea waywayandensis* DSM44232, obtained from the DSMZ (Braunschweig, Germany) collection and *Amycolatopsis mediterranei* JCM4789, obtained from the NRRL (Peoria, IL, USA) collection (NRRL B-3240T) were used as positive controls for PLA degradation [23,24]. The thermophilic strain *Amycolatopsis viridis* (NRRL B-24837T) was used as a positive control for growth at 45 °C [22]. All other bacterial strains used were isolated in this work.

#### 2.2.2. Media

International Streptomyces Project Synthetic Salts-Starch Medium (ISP4) (10 g L^−1^ starch, 1 g L^−1^ K_2_HPO_4_, 1 g L^−1^ MgSO_4_ · 7H_2_O, 1 g L^−1^ NaCl, 2 g L^−1^ (NH_4_)_2_SO_4_, 2 g L^−1^ CaCO_3_, 0.1 mg L^−1^ FeSO_4_ · 7H_2_O, 0.1 mg L^−1^ MnCl_2_ · 4H_2_O, 0.1 mg L^−1^ ZnSO_4_ · 7H_2_O, 15 g L^−1^ agar) was used for actinomycete cultivation. ISP4M was obtained from ISP4 by replacing starch with skim milk (10 g L^−1^), and cycloheximide (20 mg L^−1^) was added to prevent fungal growth. ISP4M was used to isolate actinomycetes from the soil samples.

GPHF (10 g L^−1^ glucose, 5 g L^−1^ peptone, 5 g L^−1^ yeast extract, 5 g L^−1^ beef extract, 0.74 g L^−1^ CaCl_2_ 2H_2_O) was used to test the ability of actinomycete strains to grow at different temperatures.

Basal medium (BM) and soil extract medium (SEM) were used to detect PLA biodegradation. BM was prepared as described by Kim and Park [17] with some modifications (the yeast extract concentration was increased from 0.06 to 0.1 g L^−1^, and CrCl_2_ was omitted): 2.34 g L^−1^ K_2_HPO_4_, 1.33 g L^−1^ KH_2_PO_4_, 0.2 g L^−1^ MgSO_4_ · 7H_2_O, 1 g L^−1^ (NH_4_)_2_SO_4_, 0.5 g L^−1^ NaCl, 0.1 g L^−1^ yeast extract, and 1 mL of trace element solution containing 11.9 mg of CoCl_2_, 11.8 mg of NiCl_2_, 15.7 mg of CuSO_4_, 0.97 g of FeCl_3_, 0.78 g of CaCl_2_, and 10 mg of MnCl_2_ per liter of deionized water. The pH of the medium was adjusted to 7, and 0.1% (*w*/*v*) gelatin was added to the medium if required by the experiment.

The SEM was obtained by stirring 200 g of sieved soil in 300 mL of water for 15 min. The obtained soil suspension was sterilized by autoclaving for 20 min and subsequently centrifuged at 700× *g* for 20 min. The supernatant was recovered and filtered through Whatman filter paper (Merck, Milan, Italy). Depending on the experiment, 0.1% (*w*/*v*) gelatin was added or not to the filtrate. The filtrate with or without gelatin was then sterilized by autoclaving.

PLA emulsified in BM was obtained by first dissolving 0.1 g of PLA in 10 mL of dichloromethane for 2 h; then, the obtained solution was added to 100 mL of 10^−8^ M sodium dodecyl sulfate (SDS) and blended by the ultrasonic disintegrator Soniprep 150 (MSE, Heathfield, UK) at maximum power four times (5 min each) with intermittent cooling on ice (1 min) after each sonication cycle. After emulsification, the solution was maintained at 80 °C for 1 h to remove dichloromethane by evaporation. Then, the components of BM (0.234 g of K_2_HPO_4_, 0.133 g of KH_2_PO_4_, 0.02 g of MgSO_4_, 0.1 g of (NH_4_)_2_SO_4_, 0.05 g of NaCl, 0.01 g of yeast extract, 0.1 mL of trace element solution) were added to the solution. The medium was sterilized by autoclaving at 120 °C for 20 min. The emulsified PLA agar medium was obtained by adding 15 g L^−1^ agar to the liquid medium before sterilization.

### 2.3. Actinomycete Isolation from Soils

Twenty soils were sampled from different geographical areas in Tuscany, Italy (agricultural, forest, garden, city, and industrial sites) (Table S1). Soils were sieved to remove gravel and large organic materials. Then, to increase the frequency of actinomycetes with respect to the total bacterial population, each soil sample was subjected to one of following treatments: (i) 5 g of soil was dried at 120 °C for 1 h [25]; or (ii) 5 g of soil was moistened with 2 mL of distilled sterile water and subsequently irradiated by microwaves (120 W for 3 min) in a water bath, as described by Wang et al. [26]. To detach the bacteria from the soil particles, the pretreated soil samples (5 g) were suspended in 50 mL of distilled water and homogenized in a Waring blender 8010ES (Christison, Newcastle upon Tyne, UK) three times (1 min each) at low speed with intermittent cooling on ice after each minute. The supernatants obtained from the soil treatments were serially diluted to 10^−4^ in sterile 0.8% NaCl. From each dilution, 100 µL was spread over the surface of ISP4M mixed with cycloheximide (20 mg L^−1^; added to prevent fungal growth). The plates were incubated at 30 °C for 14 days. Colonies surrounded by a clarification halo on ISP4M were identified as strains producing extracellular proteases.

### 2.4. Screening of Potential PLA-Degrading Actinomycetes

Actinomycete colonies that formed a clarification halo on ISP4M were recovered and streaked over the surface of an emulsified PLA agar medium and grown at 28 °C for 14 days. Then, plates were visually inspected to detect clarification halos [19]. *L. waywayandensis* DSM44232 [20] and *A. mediterranei* NRRL B-3240T [23] were used as positive controls.

### 2.5. Identification of Potential PLA-Degrading Actinomycetes

Actinomycete isolates were grown in ISP4 agar plates for three days. Then, colonies of each strain were recovered from the agar plates with a sterile cotton swab and suspended in 1 mL of sterile 0.8% (*w*/*v*) NaCl. The bacterial suspension was maintained at 100 °C for 10 min then centrifuged at 10,000× *g* for 5 min. The supernatant containing the DNA from the lysed biomass was recovered for 16S rDNA PCR amplification (2 µL of the supernatant was used in the PCR amplification). The 16S rDNA was amplified with the eubacterial universal primer pair 63F 5′-(CAGGCCTAAYACATGCAAGTC)-3′ and 1387R 5′-(GGGCGGWGTGTACAAGGC)-3′ [27]. The reaction mixture contained 2 µL of DNA as a template, 1x reaction buffer (10 mM Tris pH 8.3, 50 mM KCl, 1.5 mM MgCl_2_), 200 μM of each dNTP, 0.1 μM of each primer, and 0.05 U of DreamTaq DNA polymerase (Thermo Scientific, Rodano, Italy). PCR conditions consisted of an initial denaturation at 94 °C for 5 min, 30 cycles at 95 °C for 30 s, 55 °C for 90 s, 72 °C for 90 s, and a final extension at 72 °C for 5 min.

The PCR amplicons were sequenced at the CIBIACI Center (University of Firenze, Florence, Italy) by a 3730XL DNA Analyzer (Applied Biosystems, Foster City, CA, USA) using the same primer set as that used in the PCR amplification. All sequences were analyzed and edited using BioEdit software [28]. All 16S rDNA sequences were compared with sequences in GenBank (https://www.ncbi.nlm.nih.gov/genbank/) by the Basic Local Alignment Search Tool (BLAST) online service [29] to determine the approximate phylogenetic position. Sequences were then aligned using ClustalW with related 16S rDNA sequences retrieved from GenBank. The evolutionary history was inferred using the neighbor-joining method [30] with a bootstrap test of 1000 replicates [31]. The evolutionary distances were computed using the maximum composite likelihood method and are expressed as the number of base substitutions per site [32]. *Saccharomonospora viridis* DSM 4301(T) was used as the outgroup. All ambiguous positions were removed for each sequence pair (the pairwise deletion option). Evolutionary analyses were conducted in MEGA X [33].

### 2.6. Growth at Different Temperatures

Strains were grown in ISP4 agar medium at 30 °C for three days. Then, 3 mL of sterilized 0.8% (*w*/*v*) NaCl was added to the plate, and the biomass was scraped with a sterile spreader. The recovered bacterial suspension (30 µL) was used to inoculate 3 mL of GPHF in microbiological tubes. Each culture was set up in triplicate and incubated at 28, 37, or 45 °C in a rotary shaker at 100 rpm for seven days. Then, cultures were visually inspected to evaluate bacterial growth. The thermophilic strain *A. viridis* NRRL B-24837(T) was used as a positive control for growth at 45 °C [34].

### 2.7. PLA Biodegradation

#### 2.7.1. Utilization of Lactic Acid as a Carbon Source

Bacterial strains were grown in ISP4 agar medium at 30 °C for three days. Then, 3 mL of sterilized 0.8% (*w*/*v*) NaCl was added to the plate, and the biomass was scraped with a sterile spreader. The recovered bacterial suspension (30 µL) was used to inoculate 3 mL of BM (pH 7), to which 0.2% (*w*/*v*) lactic acid, 0.2% (*w*/*v*) glucose (as a positive control), or no carbon source (as a negative control) was added. Cultures were incubated at 30 °C in a rotary shaker at 100 rpm for seven days then visually inspected to evaluate bacterial growth. Each experiment was performed in triplicate.

#### 2.7.2. PLA Biofragmentation

Bacterial strains were grown in ISP4 agar medium at 30 °C for three days. Then, 3 mL of sterilized 0.8% (*w*/*v*) NaCl was added to the plate, and the biomass was scraped with a sterile spreader. The recovered bacterial suspension (50 µL) was used to inoculate BM containing 0.1% (*w*/*v*) emulsified PLA, both with and without 0.1% (*w*/*v*) gelatin. The final volume of the culture was 5 mL in 50 mL tubes. Non-inoculated cultures were used as a negative control. Cultures were incubated at 30 °C in a rotary shaker at 100 rpm for seven days. Bacteria clamps were removed from the medium by gentle centrifugation at 100× *g* for 1 min, then the OD_600_ of the supernatant was measured to evaluate the turbidity due to the emulsified PLA remaining in the spent medium. Each experiment was performed in triplicate.

The lactic acid concentration in the spent broths was determined with a Varian ProStar HPLC chromatograph (Agilent Technologies, Santa Clara, CA, USA). It was equipped with a Rezex™ ROA-Organic Acid H+ (8%), LC Column 300 x 7.8 m(Phenomenex Inc., Castel Maggiore, Italy) maintained at 65 °C, with a 20 μL 151 loop for sample injection and a refractive index (RI) detector. The eluent was 0.01 N H_2_SO_4_ at a flow rate of 0.6 mL min^−1^.

#### 2.7.3. PLA Biodeterioration

##### Weight Loss of PLA Films

The PLA films were prepared by dissolving 1.6 g of PLA granules in 80 mL of dichloromethane, followed by pouring 5 mL of the solution into glass Petri plates (5 cm diameter) coated with a thin layer of silicone oil to avoid the adhesion of PLA to the glass surface. The Petri plates were loosely closed with a lid and left to stand overnight to evaporate the dichloromethane. After dichloromethane evaporation, PLA films were detached from the Petri plates. Silicone traces were removed from PLA films by immersing the films in 500 mL of deionized water with 3 mL SDS (10% *w*/*v*) and maintained at 30 °C with agitation overnight. The films were rinsed in deionized water three times and dried. The PLA films were cut to obtain strips ranging from 20 to 30 mg in weight. The exact weight of each strip was determined.

The cultures used as inocula were grown on ISP4 agar medium for three days at 30 °C. Then, 3 mL of sterilized 0.8% (*w*/*v*) NaCl was added to the plates, and the biomass was scraped with a sterile spreader to obtain a bacterial suspension. Microbiological tubes (25 mL volume capacity) containing PLA film strips and 8 mL of four different media (BM, BM with 0.1% (*w*/*v*) gelatin, SEM, or SEM with 0.1% (*w*/*v*) gelatin) were sterilized by autoclaving, then inoculated with the bacterial suspension to obtain OD_600_ = 0.05. Control experiments were set up with non-inoculated media. Tubes were incubated at 30 °C at 100 rpm in a rotary shaker. After 30 days, PLA films were recovered from the medium filtering cultures through a sieve (aperture size: 600 µm) to separate the liquid cultures from the PLA films. Bacteria were detached from the film by gently washing with water to avoid film fragmentation. The weight of each PLA film was measured after drying at 60 °C for 3 h. Each experiment was performed in quadruplicate.

##### Effect of Biodeterioration on the Physical Features of PLA Film: Visual Inspection and Environmental Scanning Electron Microscopy (ESEM) Analysis

The cultures used as inocula were grown on ISP4 agar medium for three days at 30 °C. Then, 3 mL of sterilized 0.8% (*w*/*v*) NaCl was added to the plates, and the biomass was scraped with a sterile spreader. The bacterial suspension was recovered and used to inoculate two 100 mL flasks containing 50 mL of SEM + 0.1% (*w*/*v*) gelatin and PLA film (0.1 g, 5 mm diameter), prepared as described above. The initial OD_600_ was 0.05. Control experiments were set up with non-inoculated media. Flasks were incubated at 30 °C in a rotary shaker at 100 rpm. After 30 days of incubation, films were recovered from the flask. A PLA film from one flask was used for ESEM observation. The PLA surfaces with growing cultures were carefully cut out and mounted on aluminum stubs using double-sided adhesive tape and sputter-coated for 1 min at 40 mA with a thin layer of gold. ESEM imaging of the samples was performed at the Center of Electronic Microscopies (CeME, Sesto Fiorentino, Italy) by Gaia 3 (Tescan s.r.o, Brno, Czech Republic) using a 10-kV electron beam operating in high-vacuum mode and with a secondary electron detector.

The PLA film from the second flask was washed with deionized water, and the biomass was gently removed from the surface. After drying, the film was visually inspected to observe macroscopic modifications in the material (change in color, formation of holes and cracks).

To quantify the loss of transparency of the PLA films, grayscale images of the film laying on a black background were acquired and subsequently analyzed by the Analysis Toolbox module of the ImageQuant software TL VL003.02 (GE Healthcare Life Sciences, Buckinghamshire, UK). Five squares with areas of 64 pix were randomly chosen over the film surface, and the average pixel intensity was calculated. Pixel intensity ranged from 0 (white) to 255 (black). An estimation of film opacity was obtained by the Opacity Index (OI) and calculated by subtracting the average pixel intensity of the film (PIf) from the average pixel intensity of the black background (PIb).

#### 2.7.4. Statistical Analysis

Data obtained from the experiments testing the PLA degradative capabilities of the strains were statistically analyzed using R (http://r-project.org). Data were evaluated for normality with the Shapiro‒Wilk test. Parametric (ANOVA) and nonparametric (Kruskal‒Wallis) statistical tests were used for normally distributed data and non-normally distributed data, respectively. *Post hoc* tests (Bonferroni’s and Dunnett’s, respectively) were performed for normally distributed data and non-normally distributed data.

## 3. Results and Discussion

### 3.1. Isolation of Actinomycetes able to Degrade Emulsified PLA

PLA-degrading bacteria are rare in soil [5], and most of the strains isolated to date have been rare actinomycetes mainly from the families Pseudonocardiaceae, Thermomonosporaceae, Micromonosporaceae, and Streptosporangiaceae [20] (Table 1).

**Figure 1:**
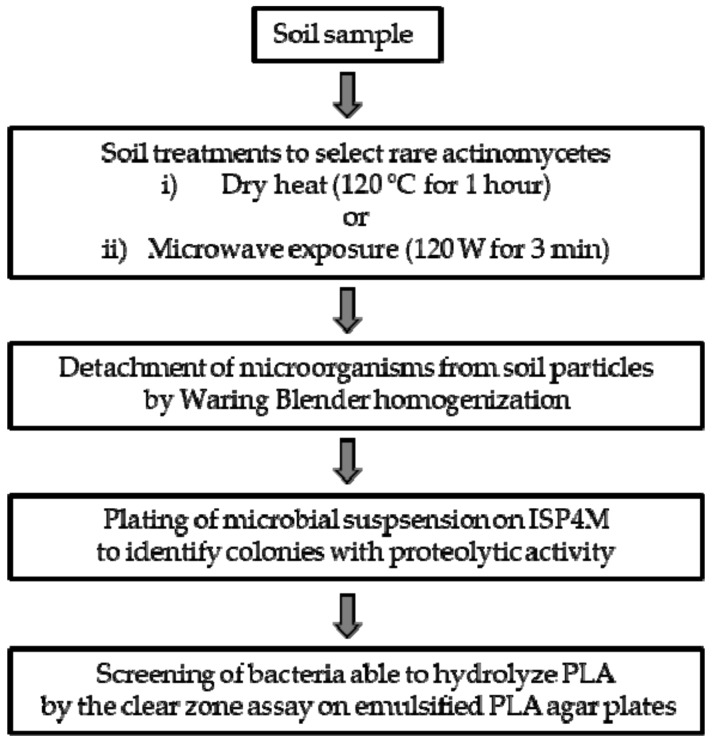
Scheme of the method used to isolate rare actinomycetes with extracellular proteolytic activity and able to hydrolyze emulsified PLA.

Many procedures to isolate the desirable rare actinomycetes from natural habitats have been developed by combining different physical/chemical pretreatments of the natural samples to eliminate unwanted microorganisms, followed by culturing on selective media to enhance the growth of rare actinomycetes [35]. In this work the probability of isolating PLA-degrading rare actinomycetes from the soil was increased by applying dry heat or microwave irradiation [25]. Subsequently, the isolation of strains has been performed on a selective medium in which skim milk was added as a carbon source instead of starch (Figure 1). Briefly, 20 soils sampled in Tuscany from agricultural, forest, garden, city, and industrial sites (Table S1) were dried at high temperature (120 °C for 1 h) or treated with microwave irradiation. Then, the bacteria were detached from soil particles by Waring blender (Waring Commercial, Torrington, CT, USA) homogenization and spread on ISP4M. Skim milk is generally used in agar plates to detect the extracellular proteolytic activity of microorganisms since colonies secreting protease form a clarification halo from the hydrolysis of milk protein [36]. It is well known that actinomycetes capable of PLA degradation generally use proteases for PLA depolymerization [37]. Eight-hundred colonies grown on ISP4M-producing clarification halos were selected and subsequently streaked over emulsified PLA agar medium to confirm their ability to hydrolyze PLA. Among the tested isolates, four were able to depolymerize PLA, as evidenced by colonies surrounded by a clarification halo. The four strains were named SO1.1 and SO1.2 (both obtained from an industrial site soil and treated at 120 °C for 1 h), SNC (isolated from soil close to a city road and treated with microwave irradiation), and SST (obtained from a garden soil treated with microwave irradiation). In summary, the method used in this work allowed us to retrieve four PLA-hydrolyzing strains from a screening of 20 soils, thereby reducing the number of soil samples analyzed relative to previous works.

### 3.2. Actinomycete Identification and Growth at Different Temperatures

Isolates were identified by 16S rRNA gene sequencing. The 16S rRNA sequences of strains SO1.1, SO1.2, SNC, and SST were deposited at GenBank under the accession numbers MN014059, MN014060, MN014061, and MN014062, respectively. All the 16S rDNA sequences showed the highest similarity to the sequences of strains belonging to the genus *Amycolatopsis*. This result further emphasizes the known importance of the genus *Amycolatopsis* in PLA biodegradation. *Amycolatopsis* strain HT-32 was the first actinomycete isolate identified as able to degrade PLA [16]; since then, quite a number of *Amycolatopsis* strains have been identified as PLA degraders [38,39,40].

**Figure 2:**
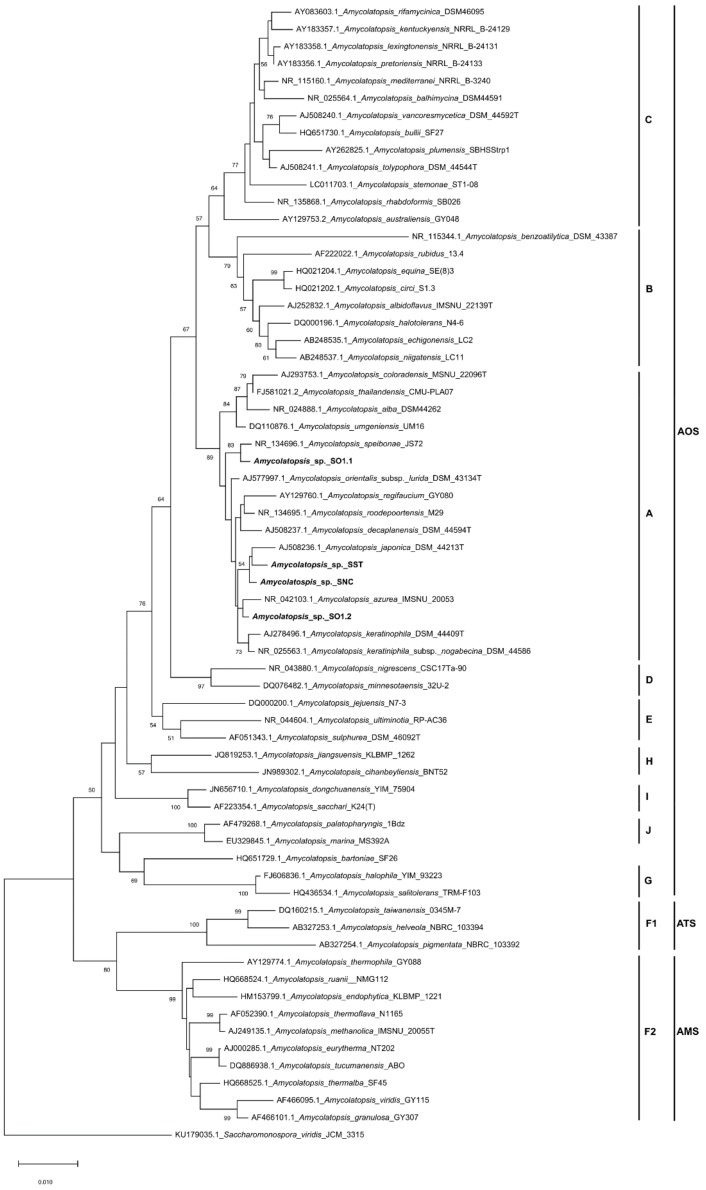
Neighbor-joining phylogenetic tree based on the 16S rDNA sequences of strains SO1.1, SO1.2, SST, SNC, and an additional 61 *Amycolatopsis* spp. strains. The scale bar indicates 10 nucleotide substitutions per 1000 nucleotides, and the number at the nodes is the percentage bootstrap value based on 1000 resampled datasets. Bootstrap values below 50% are not reported. The mesophilic or moderately thermophilic *A. orientalis* subclade (AOS); the thermophilic *A. methanolica* subclade (AMS); and the mesophilic *A. taiwanensis* subclade (ATS) are indicated, as well as groups A‒J.

The 16S rRNA gene sequences were used to build a phylogenetic tree to evaluate the phylogenetic relationships between our isolates and other *Amycolatopsis* strains (Figure 2). *S. viridis* DSM 4301(T) was used as an outgroup. *Amycolatopsis* species are grouped into three major subclades: the mesophilic or moderately thermophilic *A. orientalis* subclade (AOS); the thermophilic *A. methanolica* subclade (AMS); and the mesophilic *A. taiwanensis* subclade (ATS) [41,42]. The AOS subclade is formed by nine groups designated A‒E and G‒J, while the ATS and AMS subclades are formed by groups F1 and F2, respectively [41,43].

The phylogenetic tree associated the strains isolated here (SO1.1, SO1.2, SNC, and SST) with group A of the AOS subclade, which is known to contain mesophilic and moderately thermophilic strains. The results of the experiments that evaluated the ability of the strains to grow at 28, 37, and 45 °C showed that all four strains were able to grow at 28 and 37 °C, but not at 45 °C (data not shown), suggesting that the strains are mesophilic members of the AOS clade.

### 3.3. Degradation of PLA

The ability of the four strains SO1.1, SO1.2, SNC, and SST to degrade PLA was investigated, and their contributions to the different phases of the biodegradation process were explored [4]. *A. mediterranei* NRRL B-3240T and *L. waywayandensis* DSM 44232 were used as positive controls for PLA degradation.

#### 3.3.1. Biofragmentation

The biofragmentation capability of the bacterial strains was evaluated by measuring their ability to cleave emulsified PLA. Emulsified PLA confers turbidity to BM, and a decrease in turbidity after bacterial growth suggests that bacteria hydrolyze the PLA. This method can only be applied to strains growing in clumps, since bacterial aggregates can be easily removed from the spent medium by gentle centrifugation (100× *g* for 1 min) without causing the sedimentation of emulsified PLA. For this reason, *A. mediterranei* NRRL-3240T, which grows as a disperse mycelium, was not included in the experiment. Since PLA biofragmentation is improved by protein inducers of PLA depolymerases (e.g., gelatin, silk fibroin, and elastin) [2,44], cultures were set up both with and without 0.1% gelatin.

Table 2 reports the percentage of turbidity of the spent medium of *Amycolatopsis* strains relative to the turbidity detected in the negative control (the non-inoculated medium). The results show that both the positive control strain *L. waywayandensis* DSM 44232 and the three *Amycolatopsis* strains SO1.1, SO1.2, and SST needed 0.1% gelatin for the induction of biofragmentation activity. On the contrary, the strain SNC cleaved PLA efficiently in the presence or absence of gelatin (Table 2), suggesting that the activity of the PLA depolymerases in this strain is independent of induction by gelatin.

The strains SNC and *L. waywayandensis* DSM44232 cultured in PLA (emulsified BM with 0.1% gelatin) showed an accumulation of lactic acid in the medium (20 mg L^−1^) that exceeded that found in the non-inoculated medium (10 mg L^−1^) (data not shown), which suggests that these strains produced extracellular hydrolytic enzymes that are able to release lactic acid from the polymer. On the contrary, the strains SO1.1, SO1.2, and SST did not show a greater accumulation of lactic acid in the medium compared with the negative control (data not shown); thus, it is likely that their enzymes only produce oligomers from PLA.

#### 3.3.2. Assimilation

The ability to assimilate l-lactic acid released by depolymerized PLA is required for the complete mineralization of the polymer. The ability of the strains to use l-lactic acid as a carbon/energy source was evaluated by comparing the growth on l-lactic acid with the growth on d-glucose. All four *Amycolatopsis* strains were able to grow efficiently on glucose but showed a different growth ability in the presence of l-lactic acid (Table 3). SO1.2, SNC, SST, and *L. waywayandensis* DSM44232 grew in the presence of l-lactic acid, while SO1.1 and *A. mediterranei* NRRL B-3240T did not. Of the strains evaluated, the strain SNC had the greatest ability to use l-lactic acid as a sole carbon and energy source.

#### 3.3.3. Biodeterioration

The biodeterioration of solid PLA films by the strains was evaluated by measuring the weight loss of PLA films submerged in the bacterial cultures after one month of growth in BM or SEM with or without 0.1% gelatin. The choice of SEM was motivated by the possibility of employing the strains for the PLA degradation in soil. Since the strains grew as a biofilm on the PLA film, the determination of the film’s weight after bacterial growth required the detachment of bacteria from the polymer, but complete removal could not always be accomplished. That could lead both to an overestimation of the residual film weight and a reduction in the reproducibility of the method. In spite of these limitations, the method allowed us to identify strains that are able to induce the deterioration of PLA films (Table 4). Only two strains caused a significant weight loss in the film: the well-known PLA degrader *L. waywayandensis* DSM44232 [23], which reduced the weight of the film by about 37% in BM + 0.1% gelatin, and our isolate SNC, which induced a weight loss of about 36% in SEM + 0.1% gelatin. The inability of SNC to reduce the film weight in SEM without gelatin is in apparent contrast to the biofragmentation results, which indicated that the PLA depolymerization activity of the strain SNC does not depend on the induction by gelatin. It can be hypothesized that gelatin, even if not required for the induction of PLA depolymerization, could enhance the expression level and increase the efficiency of PLA depolymerase in this strain. At any rate, it cannot be ruled out that gelatin might have a further role to play in the biodeterioration process, and this remains to be clarified. Interestingly, the ability of the strain SNC to deteriorate PLA also depended on the growth medium; in fact, SNC was able to reduce the weight of the film in SEM + 0.1% gelatin, but not in BM + 0.1% gelatin. That indicates nutrient availability has a crucial role in the bacterial ability to deteriorate PLA. Understanding the environmental conditions that maximize the ability of the strain to deteriorate PLA (e.g., nutrient availability, the presence of PLA depolymerization inducers) is crucial for the development of biotechnological applications [37].

Overall, analysis of the biodegradation assays showed that strains positive for the clarification zone test and able to cleave PLA in its emulsified form (i.e., SO1.1, SO1.2, SST, NRRL B-3240T) were not able to significantly decrease the weight of the PLA film.

**Figure 3:**
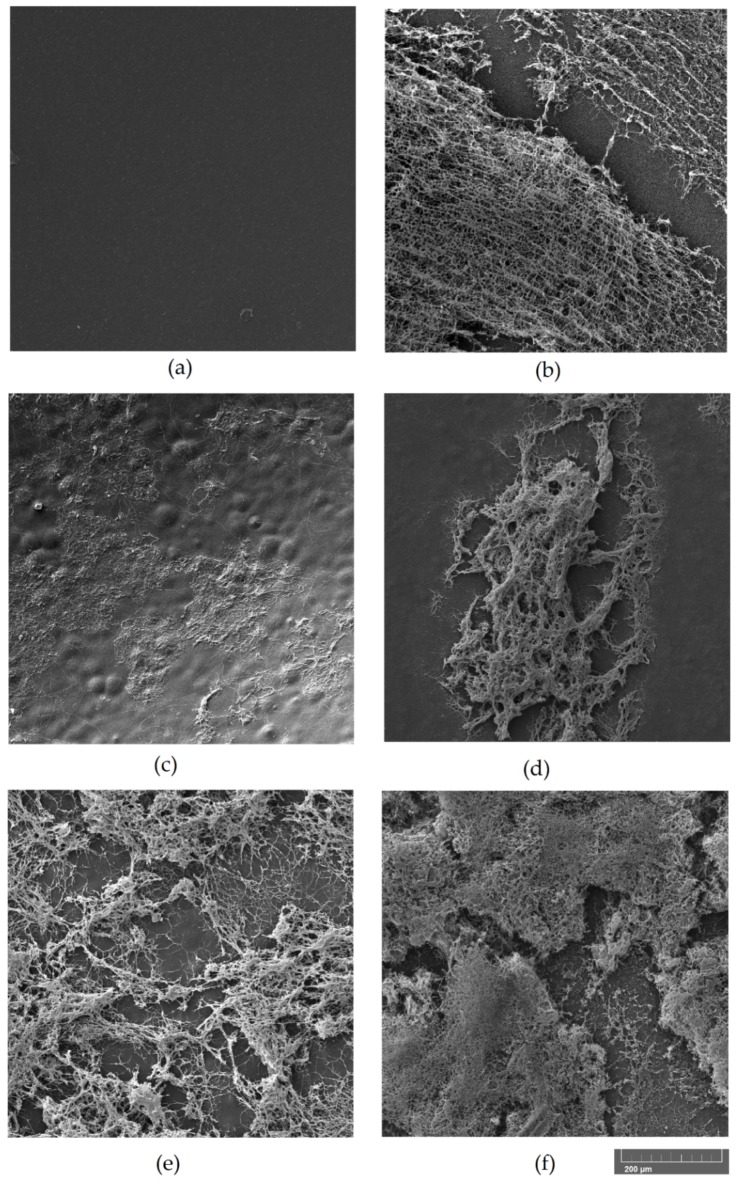
Environmental Scanning Electron Microscopy (ESEM) images of bacterial strains adhering to PLA films. Scale bar: 200 µm. (**a**) No bacteria; (**b**) *L. waywayandensis* DSM44232; (**c**) *Amycolatopsis* sp. SO1.1; (**d**) *Amycolatopsis* sp. SO1.2; (**e**) *Amycolatopsis* sp. SST; (**f**) *Amycolatopsis* sp. SNC. PLA films were incubated for one month with the bacterial strains in soil extract medium (SEM) + 0.1% gelatin.

**Figure 4:**
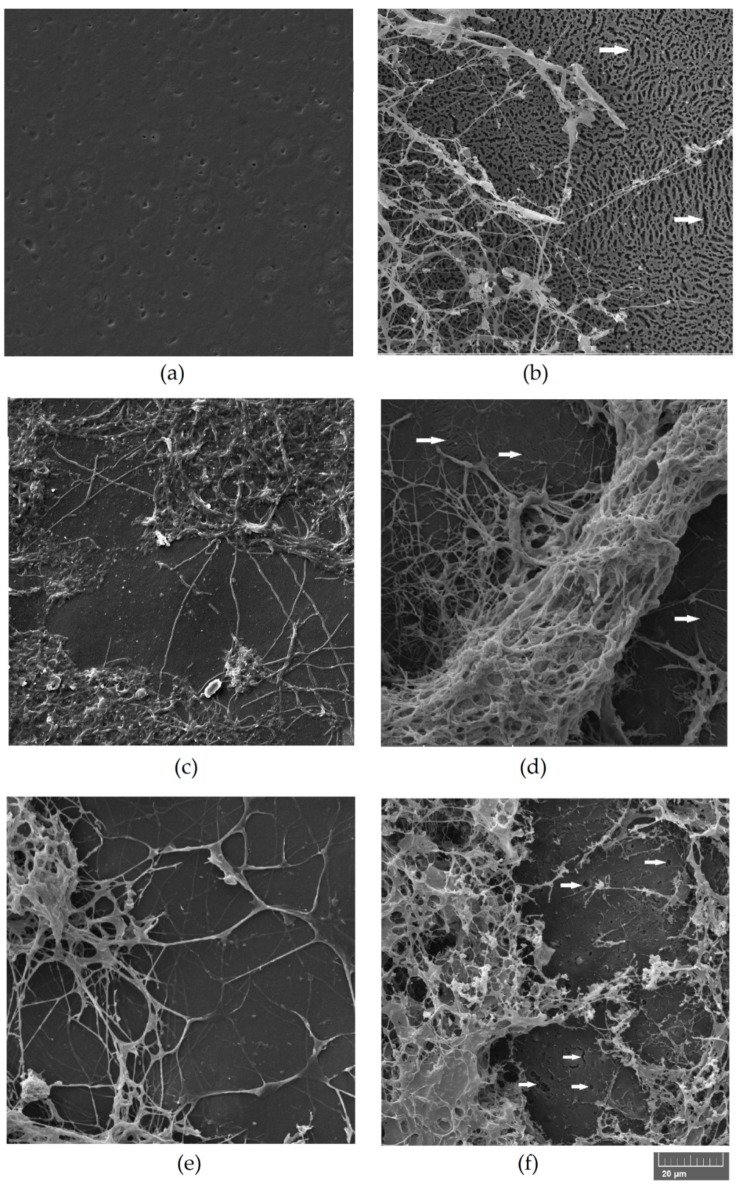
Environmental Scanning Electron Microscopy (ESEM) images of bacterial strains adhering to PLA films. Scale bar: 20 µm. (**a**) No bacteria; (**b**) *L. waywayandensis* DSM44232; (**c**) *Amycolatopsis* sp. SO1.1; (**d**) *Amycolatopsis* sp. SO1.2; (**e**) *Amycolatopsis* sp. SST; (**f**) *Amycolatopsis* sp. SNC. White arrows indicate representative holes and cracks. PLA films were incubated for one month with the bacterial strains in soil extract medium (SEM) + 0.1% gelatin.

**Figure 5:**
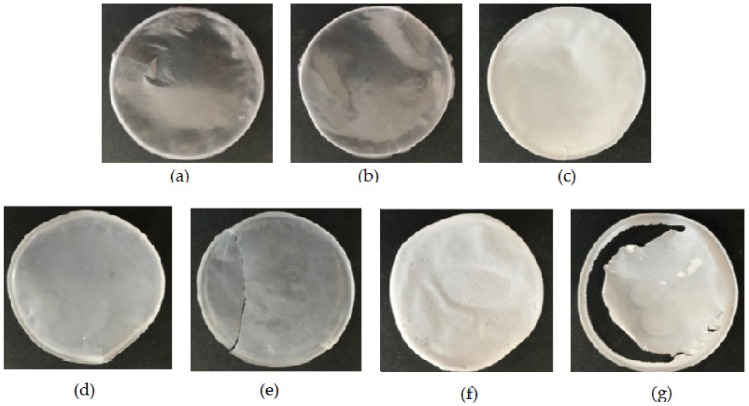
PLA films maintained for one month submerged in bacterial culture in soil extract medium (SEM) + 0.1% gelatin after the removal of attached biomass. (**a**) No bacteria; (**b**) *A. mediterranei* NRRL B-3240T; (**c**) *L. waywayandensis* DSM 44232; (**d**) *Amycolatopsis* sp. SO1.1; (**e**) *Amycolatopsis* sp. SO1.2; (**f**) *Amycolatopsis* sp. SST; (**g**) *Amycolatopsis* sp. SNC.

**Figure 6:**
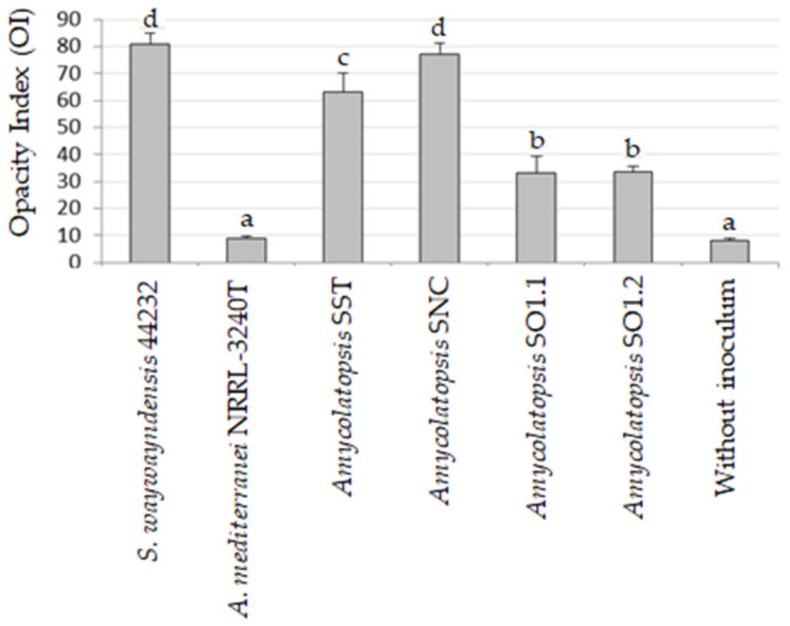
The Opacity Index (OI) of PLA films maintained for one month submerged in bacterial culture in soil extract medium (SEM) + 0.1% gelatin after removal of attached biomass. Values represent average OI ± standard deviation. Bars marked with different letters are significantly different (Bonferroni’s test, *p* < 0.01).

The biodeterioration of plastic polymers involves not only enzymatic activity but also physical and chemical deterioration; physical deterioration occurs through the formation of microbial biofilms that secrete extracellular polymeric substances (EPSs), which penetrate the pores of the polymer and induce cracks. Chemical deterioration is the result of organic acids released by the bacteria that decrease the pH and thereby induce the progressive degradation of the polymer [45]. Moreover, the ability to form abundant biofilm on the surface of PLA film could enhance the degradation process by keeping secreted hydrolytic activities localized [46]. The microscopic analysis of film surface has been utilized to confirm the degradations of PLA [3,16]. Biofilm formation over the surface of PLA film and possible alterations of the polymer were evaluated by ESEM analysis of the PLA film after bacterial growth. The results of the ESEM analysis of PLA films incubated for one month with the bacterial strains in SEM + 0.1% gelatin are reported in Figure 3 and Figure 4. All the tested strains were able to adhere and grow on PLA film, exceptof the strain *A. mediterranei* NRRL-B3240T (data not shown). Strain SO1.1 grew slightly on the PLA film (Figure 3c), and SO1.2 formed a biofilm only in some zones of the PLA film (Figure 3d), while a pseudomycelium adhering to the entire film surface was observed for the strains *L. waywayandensis* DSM 44232, SST, and SNC (Figure 3b,e,f). SNC showed a very compact and thick biofilm spread over the surface of the PLA film (Figure 3f). The same picture at higher magnification shows that both SNC and *L. waywayandensis* DSM 44232 strains produced cracks throughout the PLA film (Figure 4b,f). Cracks are also visible in some zones of the PLA film colonized by SO1.2 (Figure 4d). Visual inspection and calculation of the Opacity Index (OI) of PLA films after the removal of the attached microbial biomass were carried out to detect macroscopic holes and alterations of the transparency (Figure 5 and Figure 6). All the films incubated with microbial strains, except the strain *A. mediterranei* NRRL-B3240T, showed macroscopic alterations, while films incubated in a non-inoculated medium (the negative control) maintained their integrity and transparency (Figure 5). The OI calculated for PLA films incubated with the different strains showed that *A. mediterranei* NRRL-B3240T did not induce a significant modification of the transparency in accordance with the inability of the strain to adhere and grow over the film surface, as shown by ESEM analysis. All the other strains induced a significant increase in the opacity of the film as compared with the negative control with the following order SO1.1 = SO1.2 < SST < SNC = *L. waywayndensins* DSM 44232 (Figure 6). Strains SO1.1 and SO1.2 induced a partial loss of transparency in the films, while the strains *L. waywayandensis* DSM 44232, SST, and SNC made the films opaque and white, indicating a reduction in the molecular weight of the PLA polymer [2]. The strain SNC induced an extensive erosion of the film (Figure 6g); this observation is in accordance with the high weight loss of the film after incubation with this strain, as described above.

Results obtained from experiments that aimed to value the ability of the strains to deteriorate PLA showed that the clear zone test must be used only for preliminary screening to detect potential PLA degraders and that the actual ability of the strains to induce effective degradation of solid PLA requires further evaluation using additional tests [47]. Even if a strain is able to cleave the PLA polymer, it can still lack the mechanisms required for the biodegradation of PLA films [48]. That was especially true for *A. mediterranei* NRRL-B3240T, previously known as positive to the clear zone test [24], which was unable to adhere to the solid PLA film, induce any macroscopic modification of the polymer, or reduce the weight of the film.

## 4. Conclusions

In this work, an efficient method for the isolation of PLA-degrading bacteria from soil was developed. The method allowed us to isolate four potential mesophilic PLA-degrading bacteria belonging to the genus *Amycolatopsis*. The strain SNC was found to be an efficient PLA degrader (PLA at high molecular weight ranged from 190 to 230 KDa), since it was able to (i) assimilate lactic acid, (ii) fragment the PLA chain by hydrolytic enzymes, independently of the induction of a proteic substance (in this case, gelatin), (iii) form an extensive biofilm on the surface of PLA, and (iv) erode and significantly reduce the weight of the polymer. The other three isolates showed partial PLA degradation ability, and therefore could be useful in co-cultures with the SNC isolate.

Several studies on PLA degradation have been conducted at temperatures above 40 °C but, in nature, PLA is mostly degraded by mesophilic bacteria. Therefore, as previously reported, mesophilic bacteria are more useful than thermophilic bacteria for the biodegradation of PLA residues [7]. Since the degradative capabilities of the strain SNC were observed in mesophilic conditions (30 °C) typical of a soil environment, and SEM containing soil extracts with the addition of gelatin allowed the strain to efficiently deteriorate PLA film, SNC should be evaluated in bioaugmentation biotechnological processes that aim to biodegrade the PLA polymer in different environments.

## Figures and Tables

**Table 1:** The number of bacteria positive for the clear zone test on emulsified PLA agar plates isolated from soil.

Study	Number of Soils Used	Number of Bacteria Positive for the Clear Zone Test
Tomita et al. 2004 [14]	153	1
Teeraphatpornchai et al. 2003 [21]	400	4
Sukkhum et al. 2009 [20]	80	13
Nakamura et al. 2001 [18]	300	2
Pranamuda et al. 1997 [16]	45	1
Kim and Park 2010 [17]	60	1

**Table 2:** Percentage of residual turbidity of Basal medium (BM) containing emulsified PLA after bacterial growth. The residual turbidity of BM with emulsified PLA after the removal of bacterial clumps was evaluated for each strain after one week of growth (*n* = 3). Values represent the average percentage of residual turbidity of the spent medium relative to the turbidity of the non-inoculated medium ± standard deviation.

	BM	
Strain	without Gelatin	with 0.1% Gelatin
*Lentzea waywayandensis* DSM 44232	NS ^a^	7.2 ± 7.2 *
*Amycolatopsis* sp. SST	NS	27.9 ± 9.9 *
*Amycolatopsis* sp. SNC	6.7 ± 2.7 *	11.8 ± 6.7 *
*Amycolatopsis* sp. SO1.2	NS	11.0 ± 13.5 *
*Amycolatopsis* sp. SO1.1	NS	40.4 ± 21.8 *

^a^ No significant differences were detected. * Significant differences between the turbidity of the spent medium and the non-inoculated medium were detected (Bonferroni test, *p* < 0.05).

**Table 3:** Bacterial utilization of lactic acid as a carbon source. Bacteria were cultured in Basal medium (BM) with 0.2% (*w*/*v*) glucose or 0.2% (*w*/*v*) lactic acid. The bacterial growth was qualitatively evaluated visually.

Strain	Carbon Source	
	d-Glucose	l-Lactic Acid
*L. waywayandensis DSM 44232*	+++	+
*Amycolatopsis mediterranei NRRL B-3240T*	+++	-
*Amycolatopsis* sp. SNC	+++	++
*Amycolatopsis* sp. SST	+++	+
*Amycolatopsis* sp. SO1.1	+++	-
*Amycolatopsis* sp. SO1.2	+++	+

+++ = high growth, ++ = medium growth, + = low growth, - = no growth.

**Table 4:** Percentage of weight loss of PLA film after incubation with bacterial strains in both Basal medium (BM) and soil extract medium (SEM). Differences in the weight of the PLA films before and after incubation with bacterial strains for one month were evaluated (*n* = 5). Values represent average percentages of weight loss ± standard deviation.

	BM		SEM	
Strain	without Gelatin	with 0.1% Gelatin	without Gelatin	with 0.1% Gelatin
*L. waywayandensis* DSM 44232	NS ^a^	37.3 ± 19.4 *	NS	NS
*A. mediterranei* NRRL B-3240T	NS	NS	NS	NS
*Amycolatopsis* sp. SST	NS	NS	NS	NS
*Amycolatopsis* sp. SNC	NS	NS	NS	36.0 ± 7.3 *
*Amycolatopsis* sp. SO1.2	NS	NS	NS	NS
*Amycolatopsis* sp. SO1.1	NS	NS	NS	NS
Without inoculum	NS	NS	NS	NS

*^a^ No significant differences were detected. * Significant differences (Bonferroni test, *p* < 0.01) between the percentage of weight loss detected in the inoculated films and non-inoculated films.

## Supplementary Materials

The following are available online at https://www.mdpi.com/2076-2607/7/12/590/s1, Table S1.
