# Supplementary material for: The Degradative Capabilities of New Amycolatopsis Isolates on Polylactic Acid
Source: Microorganisms. 2019 Nov 20;7(12):590. doi: 10.3390/microorganisms7120590 (PMC6955660; doi:10.3390/microorganisms7120590)
Supplement: Supplementary file 1 [file microorganisms-07-00590-s001.pdf]

Table S1. Soil samples used in this work.

Sample name	Source	Sampling site	Sampling date
SCA1	Agricultural soil	Signa (FI)	29/10/2017
SGF1	Garden soil	Carmignano (PO)	22/09/2017
SGN1	Grassland soil in idustrial site	Sesto Fiorentino (FI)	1/3/2017
SL1	Forest soil	Lentula (PT)	1/11/2017
SL2	Forest soil	Lentula (PT)	1/11/2017
SL4	Forest soil	Lentula (PT)	1/11/2017
SL5	Forest soil	Lentula (PT)	1/11/2017
SL6	Forest soil	Lentula (PT)	1/11/2017
SNC1	Grassland soil in city site	Gambassi (FI)	1/11/2017
SO1	Grassland soil in idustrial site	Sesto Fiorentino (FI)	29/10/2017
SO2	Grassland soil in idustrial site	Sesto Fiorentino (FI)	29/10/2017
SPO1	Grassland soil in idustrial site	Sesto Fiorentino (FI)	21/11/2017
SST1	Graden soil	Firenze	15/11/2017
SST2	Agricultural soil	Campi Bisenzio (FI)	15/11/2017
STC1	Agricultural soil	Pistoia	18/05/2017
STM1	Forest soil	Castelnuovo Garfagnana (LU)	2/10/2017
STM2	Forest soil	Castelnuovo Garfagnana (LU)	2/10/2017
STM3	Forest soil	Castelnuovo Garfagnana (LU)	2/10/2017
STNT1	Soil from greenhouse	Pistoia	18/05/2017
STV1	Soil from greenhouse	Pistoia	18/05/2017
